# Creation of an Open-Access High-Resolution Tandem Mass Spectral Library of 1000 Food Toxicants

**DOI:** 10.1021/acs.analchem.5c03020

**Published:** 2025-10-10

**Authors:** Federico Padilla-González, Serena Rizzo, Caroline Dirks, Wout Bergkamp, Sjors Rasker, Ivan Aloisi

**Affiliations:** Wageningen Food Safety Research, 4508 Wageningen University & Research, Akkermaalsbos 2, Wageningen 6708 WB, The Netherlands

## Abstract

Spectral library searching is a key method for compound annotation in mass spectrometry; however, existing libraries often suffer from high data heterogeneity, varying spectral quality, or limited accessibility. These issues are particularly significant in food safety, where the lack of comprehensive reference data hampers the identification of hazardous compounds. To address these limitations, we developed the WFSR Food Safety Mass Spectral Library, a freely accessible tandem mass spectral library focused on food contaminants, residues, and hazardous compounds. This library contains 6993 manually curated spectra from 1001 compounds acquired in positive ionization mode using ultrahigh-performance liquid chromatography coupled to an Orbitrap IQ-X Tribrid mass spectrometer. Spectra were recorded at seven collision energies under standardized conditions. Comprehensive metadata are provided, including common names, CAS, SMILES, InChIKeys, retention times, and compound classes. The library is publicly available via a dedicated website (https://www.wur.nl/en/show/food-safety-mass-spectral-library.htm) and through the GNPS repository, adhering to FAIR data principles to facilitate community reuse. Comparisons with major repositories (GNPS, MassBank, MoNA, and MSnLib) showed that 216 compounds (22.2%) are unique to our library. Further analysis using molecular networking and MS2Query revealed that about 38% of the compounds lack reliable matches in public libraries. The WFSR spectral library is designed to improve the annotation of food toxicants and facilitate the identification of structural analogues using computational tools. This library is part of an ongoing initiative with future updates planned to include negative ionization mode spectra and an expanded compound repertoire, aiming to advance food safety monitoring.

## Graphical abstract

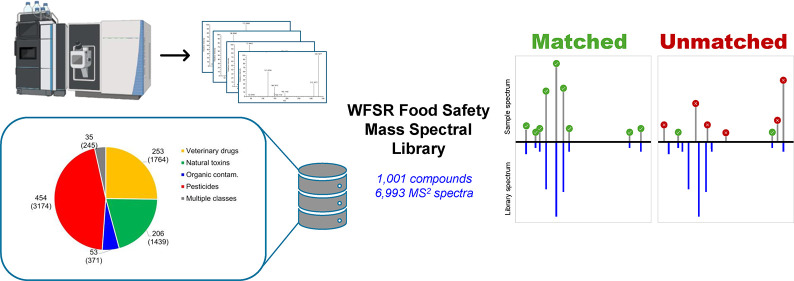

Food safety is a critical public health concern, requiring effective detection of hazardous substances in food matrices to safeguard consumers’ health. However, unambiguously identifying a wide range of chemical hazards in complex matrices remains challenging. Liquid chromatography coupled with high-resolution tandem mass spectrometry (LC-HRMS/MS) is the technique of choice for accurately detecting and identifying medium to high polar compounds, particularly in complex matrices that characterize food and environmental samples.

Unlike targeted approaches, which focus on detecting a predefined set of “targeted” compounds for which reference standards are available, untargeted LC-HRMS aims to comprehensively profile all detectable analytes in a sample without prior knowledge of their identity. However, the large amount of data generated during untargeted LC-HRMS studies requires reliable tools for compounds’ annotation and identification. While compounds’ identification often entails the comparison of spectral data and retention time values with those of reference standards, compounds’ annotation often refers to the assignment of tentative identities or descriptors to an unknown MS feature based on matching their accurate masses, tandem mass spectrum acquired at either low or high resolution (hereafter referred to as MS/MS), or isotopic pattern with database or library entries.^1,2^ Therefore, the most common approach for compound annotation is spectral library searching.^1^ In this approach, MS/MS spectra of detected features are matched to reference spectra from known molecules, allowing compounds’ annotation based on shared similarities in their MS/MS “fingerprints”.^1^ As this approach relies on the availability of reference spectra, spectral libraries applicable to LC-MS/MS have grown exponentially over the past decades from tens of thousands to millions of MS/MS spectra originating from hundreds of thousands of compounds.^1–3^

Despite their value, generating comprehensive reference spectral libraries is resource-intensive and requires extensive quality checks.^4^ Moreover, some of the largest and most widely used spectral libraries, such as NIST (https://chemdata.nist.gov/), mzCloud (https://www.mzcloud.org/), and METLIN^3^ (https://metlin.scripps.edu/)*,* are only available through commercial licenses. Fortunately, several open-access and freely accessible MS/MS spectral libraries, such as GNPS,^5^ MassBank,^6^ Massbank of North America (MoNA; https://mona.fiehnlab.ucdavis.edu/), Massbank EU (https://massbank.eu/MassBank/), the Human Metabolome Database (HMDB),^7^ and MSnLib,^8^ are publicly accessible and have grown significantly in recent years.^1^

However, while several spectral libraries exist, they are limited in their representation of food toxicants, and no dedicated, open-access, and freely accessible spectral library of food contaminants, residues, and chemical hazards (herein referred as food toxicants) currently exists. This lack of spectral data poses a significant barrier to the rapid and accurate identification of such compounds in food safety applications. For instance, the commercial METLIN library, which contains over 4,000,000 curated high-resolution tandem mass spectra from more than 850,000 reference standards,^3^ has historically focused on lipids, dipeptides, and nucleotides. However, no information regarding the identity of each of those standards or whether they include food safety-relevant compounds has been made publicly available. In contrast, the open-access GNPS libraries, containing over 500,000 MS/MS spectra, have historically focused on natural products but also include drugs, human, food, and environment-derived metabolites.^1,5^

To address this gap in data availability on food toxicants, we built a spectral library comprising 1001 compounds and 6993 HRMS/MS spectra. This library covers a wide range of compound classes monitored in food safety, including organic contaminants, natural toxins, pesticides, veterinary drugs, and several relevant metabolites. It offers unique spectral data for harmful substances that are often underrepresented or absent in publicly accessible repositories. Spectra were acquired at seven collision energies, and the data were manually curated and validated using computational tools to ensure robustness and reliability for food safety applications.

By sharing this library through open-access platforms, including a dedicated website and online repositories like GNPS, we aim to facilitate rapid identification workflows, enable high-throughput screening, and enhance food safety monitoring. Furthermore, the library supports the identification of structural analogues when integrated with other computational tools and can be used in machine learning applications to predict structures or chemical classes from unknown HRMS/MS spectra.

## Experimental Section

### Chemicals and Standards

Analytical-grade methanol (MeOH) and water (H_2_O) were used to prepare the eluents, while ammonium formate (NH_4_COOH) and formic acid (HCOOH) were employed as buffers. A complete list of the reference standard names, along with their molecular formulas, CAS numbers, SMILES and InChIKey codes, as well as experimental precursor ions (*m*/*z*), and retention times, is provided in Table S1.

### Preparation of Reference Standard Solutions

Stock solutions of standard mixtures containing between two and approximately 100 compounds were diluted in H_2_O:MeOH in varying ratios from 90:10 to 50:50 (v/v) to achieve final concentrations ranging from 50 to 500 ng/mL. In a few cases, a solvent composition outside that range was used because it was not possible to dilute the standards further. Individual injections were performed for enantiomeric and isobaric compounds to ensure accurate spectral selection during the library-building phase (see the Sublibrary Creation section). Together, these mixtures encompassed over 1000 distinct compounds.

### UHPLC-HRMS/MS Analysis

Ultrahigh-performance liquid chromatography coupled with high-resolution tandem mass spectrometry (UHPLC-HRMS/MS) was used to acquire retention time and spectral data of the target compounds. The analysis was carried out on a Vanquish Horizon UHPLC system coupled to an Orbitrap IQ-X Tribrid mass spectrometer, operating in positive ionization mode. The mass spectrometer was equipped with a heated electrospray ionization (HESI-II) source (Thermo Scientific, San Jose, CA, USA). The instrument was mass-calibrated weekly, ensuring a maximum mass deviation of 1.5 ppm. Additionally, before each injection, a one-point mass calibration was automatically performed using the internal calibration mode known as RunStart EASY-IC.

The chromatographic separation of the standard mixtures was conducted using a Waters BEH C_18_ column (2.1 mm × 100 mm, 1.7 μm particle size) at a temperature of 50 °C and a flow rate of 0.3 mL/min. The mobile phases employed for the liquid chromatography separation were (A) H_2_O and (B) MeOH, both containing 2 mM NH_4_COOH and 0.1% HCOOH. The following gradient was applied: 0% B at 0 min, followed by a linear increase to 100% B until 15 min. This was followed by a washing phase at 100% B until 21 min, then a conditioning phase back to 0% B at 22 min, and 0% B from 22 to 25 min. The injection volume was set at 5 μL for all samples, blanks, and quality controls (QCs), and the tray temperature was maintained at 10 °C.

Mass spectrometry detection was performed in positive ionization mode using the full-scan and data-dependent HRMS/MS acquisition modes. Full-scan analysis was performed at a resolution of 120,000 fwhm at *m*/*z* 200 over the range of 100–1100 *m*/*z* using a spray voltage of +3.4 kV, a vaporizer temperature of 350 °C, and an ion transfer tube temperature of 320 °C. Additional parameters for the MS included sheath gas (Arb) at 48, aux gas (Arb) at 11, sweep gas (Arb) at 2, automatic gain control (AGC) target set to “custom”, normalized AGC target (%) set to 100, maximum injection time (MIT) mode set to “auto”, and a cycle time of 0.8 s. All data were collected in centroid mode.

For each standard mixture, inclusion lists containing a maximum of 25 compounds were built for HRMS/MS data acquisition. For enantiomeric and isobaric compounds, separate inclusion lists were built, and individual injections were performed to ensure accurate spectral selection during the library-building phase. In total, 380 inclusion lists were created, each generating its own raw file. For each compound, a normalized higher-energy collisional dissociation (HCD) (NCE, %) type, with a range of energies of 15, 30, 45, 60, 75, and 90, along with stepped collision energies (SCE, %) of 25, 38, and 59, was selected to perform fragmentation. The HRMS/MS data were acquired using an isolation window of 1.6 *m*/*z* (±0.8 *m*/*z*) with a minimum intensity threshold set at 1.0 × 10^5^ and a resolution of 15,000 fwhm.

To ensure optimal chromatographic and system performance, two QC mixtures, QC1 (18 compounds) and QC2 (16 compounds), were analyzed at the beginning and/or at the end of each sequence (Table S2). These compounds comprised mostly commercial pesticides diluted at 20 ng/mL in H_2_O:MeOH (90:10 v/v). A total of 53 injections of both QC mixtures were performed during a time span of 26 injection days. These QCs were used to evaluate repeatability and consistency in mass accuracy, retention time, and signal intensity over time. Additionally, a solvent blank (H_2_O:MeOH 50:50, v/v) was injected before each standard mixture to minimize carry-over phenomena.

### Sublibrary Creation

A total of 380 raw files, generated from the UHPLC-HRMS/MS analysis of the standard mixtures or individual compounds, were imported into mzVault software (version 2.3 SP1, Thermo Fisher Scientific). These raw files were first converted into compound lists, which were then used to build spectral libraries referred to as sublibraries. For each compound in the inclusion lists, the common name, raw data file, molecular formula, and the most abundant precursor ion, for either the protonated molecule [M + H]^+^, the molecular ion [M]^+^, or an adduct ([M + Na]^+^ or [M + NH_4_]^+^), were entered into mzVault based on previous analyses. One spectrum for each unique collision energy was retrieved from the raw file; then, the “averaged spectra” option was selected to merge multiple HRMS/MS spectra from the same compound, and a noise reduction step was applied through the “spectra threshold” option. Subsequently, compounds’ metadata, including SMILES, InChIKey, ChemSpider ID, and chemical structure, were automatically retrieved from ChemSpider (https://www.chemspider.com). Sublibraries were then saved in .db format and later merged into a single file that underwent subsequent curation steps (see below).

### Spectra and Metadata Curation

The spectral library underwent meticulous manual curation to ensure data accuracy and quality. First, spectra for each collision energy were visually inspected to identify and exclude low-quality spectra. An absolute intensity threshold of 5.0 × 10^4^ was established for this purpose. Spectra below this threshold were removed and their corresponding compounds recorded in an internal list for reanalysis later in the project (data not shown). Subsequently, the metadata for each compound were individually verified to ensure that correct information was imported from ChemSpider. As the retrieval of this information was based on the compound name and molecular formula, occasional mismatches occurred, such as importing metadata for an incorrect enantiomeric or isobaric compound. To verify potential errors and correct them, a systematic workflow was implemented, as follows: (i) First, the CAS number of each of the 1001 compounds was retrieved from an internal database, as provided in the supplier documentation. These CAS numbers were searched in PubChem (https://pubchem.ncbi.nlm.nih.gov/) to remove counterions and retrieve the CAS of the corresponding parent compounds; (ii) the curated CAS numbers were then used to perform a batch search in CompTox (https://comptox.epa.gov/) to retrieve their corresponding InChIKey codes, originated from measured compounds with the right stereochemistry. These codes were then matched against the ones automatically retrieved from ChemSpider for each compound, and the 114 mismatching codes were manually interrogated, giving priority to the CompTox InChIKey, as it can be linked back to the CAS of the measured compound. From this search, 17 compounds were not included in CompTox. These compounds were manually checked, and the InChIKey associated with its CAS number was retrieved from ChemSpider or Pubchem. (iii) The final InChIKeys were then used as a search list in CompTox to retrieve the SMILES codes of the 1001 compounds. Given that pyrrolizidine alkaloids (PAs) normally have complex stereochemistries and it is not uncommon to find conflicting information in different databases, the chemical structures of these compounds as published by the Joint FAO/WHO Expert Committee on Food Additives (JEFCA) were used.^9^ The .mol file of these structures was used to calculate the SMILES and InChIKey codes in the software DataWarrior.^10^

Following this metadata curation, the spectral library in .db format was edited in the software SQLiteStudio (https://sqlitestudio.pl/) to include all the curated information. Consistency in scan numbers and retention times for each HRMS/MS spectrum was also verified, and based on the mass difference between the neutral molecule and the precursor ion, the adduct type ([M + 2H]^+^, [M + Na]^+^, and [M + NH_4_]^+^) was assigned next to the compound name for better clarity. Duplicate compounds present in multiple mixtures were removed before technical validation.

Concomitant to manual curation, the “compound class” column was populated based on four predefined classes commonly monitored in food safety laboratories: organic contaminants, natural toxins, pesticides, and veterinary drugs. This broad classification approach, rather than assigning compounds to specific chemical or bioactivity categories, was chosen to facilitate filtering for end-users interested in compounds from a particular “class” only. The final spectral library file contains 1001 unique compounds and 6993 HRMS/MS spectra.

### Data Analysis and Technical Validation

We used three complementary data analysis methods to assess the technical accuracy and representativeness of our library in comparison to other open-access spectral libraries: (1) comparison of InChIKey codes with online databases, (2) spectral matching through molecular networking, and (3) spectral matching using MS2Query software. Figure S1 shows the general workflow followed for library curation and technical validation.

We compared InChIKey codes from the compounds in our spectral library with those from several publicly available resources: the GNPS library (ALL_GNPS_NO_PROPOGATED + matchms cleaned version, downloaded on April 20, 2025 at https://external.gnps2.org/gnpslibrary), MassBank EU 2024.11 (RIKIEN.msp format, available at https://github.com/MassBank/MassBank-data/releases/tag/2024.11), Mass Bank of North America (MoNA, LC-MS/MS Positive Mode, available at https://mona.fiehnlab.ucdavis.edu/downloads), and MSnLib (all positive mode .mgf files, available at https://zenodo.org/records/13911806). Comparisons were carried out using the matchms^11,12^ package in Python 3.11, considering only the first 14 characters of the InChIKey codes (hereafter referred to as planar InChiKeys), as previously performed by Brungs et al.^8^ A merged and cleaned version of these libraries (available online)^12^ was also used to visualize the chemical space covered by our spectral library, using the ChemPlot^13^ package in Python 3.9, following the script provided in its GitHub page (https://github.com/mcsorkun/ChemPlot) and for spectral matching in MS2Query.

Molecular networking and library annotation were performed in the GNPS platform.^5^ Each of the 380 raw files (.raw format) was first converted to the open .mzML format using the MSConvert tool from the software ProteoWizard version 3.0.9798 (Proteowizard Software Foundation, Palo Alto, CA, USA). The converted files were then uploaded to GNPS (https://gnps.ucsd.edu) for classic molecular networking and library annotation. The .mzML files were submitted to the GNPS “batch annotation” workflow along with a corresponding .tsv file containing compound names, precursor masses, scan numbers for HRMS/MS spectra (NCE30), filenames, and molecular identifiers (SMILES and InChIKey), following the GNPS documentation and template spreadsheet (available at https://gnps.ucsd.edu). The resulting .mgf file was then processed by classic molecular networking,^5^ with the “MScluster” option left disabled. Data filtering involved excluding all fragment ions within ±17 Da of the precursor *m*/*z* value. Additionally, HRMS/MS spectra were window-filtered by retaining only the top four fragment ions within ±50 Da across the spectrum. The mass tolerance for precursor ions was set at 0.02 Da, as was the tolerance for fragment ions. A network was constructed with edges filtered to retain only those with a cosine score greater than 0.7 and at least four matched peaks. Furthermore, connections between nodes were preserved if each node was among the other’s top 10 most similar nodes. To control network size, the maximum molecular family size was capped at 100, and lower-scoring edges were trimmed from families exceeding this limit. The spectra within the network were subsequently matched against the spectral libraries in GNPS following the same protocol as the input data. Molecular networks were visualized using the software Cytoscape.^14^ Additional to this data analysis step, the entire library was made publicly available in GNPS (see the data availability section).

Data analysis in MS2Query^15^ (version 1.5.3) was carried out using the manually curated spectral library in .msp format, exported from mzVault, for each collision energy separately. MS2Query was executed via the command line in Python 3.9, following the script provided on its official GitHub page (available at https://github.com/iomega/ms2query). Library matching was performed using a curated and freely available spectral library containing 426,280 MS/MS “cleaned” spectra derived from GNPS, MassBank, MoNA, and MSnLib.^12^ To determine the annotation accuracy, the library’s planar InChIKeys, retention times, and *m*/*z* values were compared to the MS2Query annotations, and the results reported as percentage values.

## Results and Discussion

LC-HRMS/MS-based spectral libraries have grown exponentially over the past few decades. However, the absence of a dedicated, open-access spectral library focused on food toxicants presents a significant challenge, hindering the rapid and accurate identification of harmful substances in food safety applications. To bridge this gap, we developed the WFSR Food Safety Mass Spectral Library, a comprehensive spectral library of food contaminants, residues, and chemical hazards, freely available to the scientific community (see the data availability statement section).

### Library Description and Comparison with Other Libraries

Following data acquisition and library construction, a meticulous manual curation and technical validation process was undertaken to ensure the accuracy and quality of the spectral data. As a result, approximately 25% of the compounds initially expected to be present in the standard mixtures were either not acquired or manually excluded, yielding a final library consisting of 1001 unique compounds and 6993 HRMS/MS spectra. Future updates will prioritize the reacquisition of the missing compounds and the addition of spectra acquired in negative ionization mode to enhance the library’s comprehensiveness.

As observed in Figure 1A, nearly half of the compounds in the WFSR library are pesticides (454 unique entries). These are followed veterinary drugs (253 unique entries), natural toxins (206 unique entries), and organic contaminants (53 unique entries). This last class is less represented in the current version given that the available compounds belonging to this class (e.g., PFAS, etc.) ionize better or exclusively in negative mode. Additionally, 35 compounds have been categorized under multiple classes, as they belong to two or more of the four aforementioned categories.

**Figure 1:**
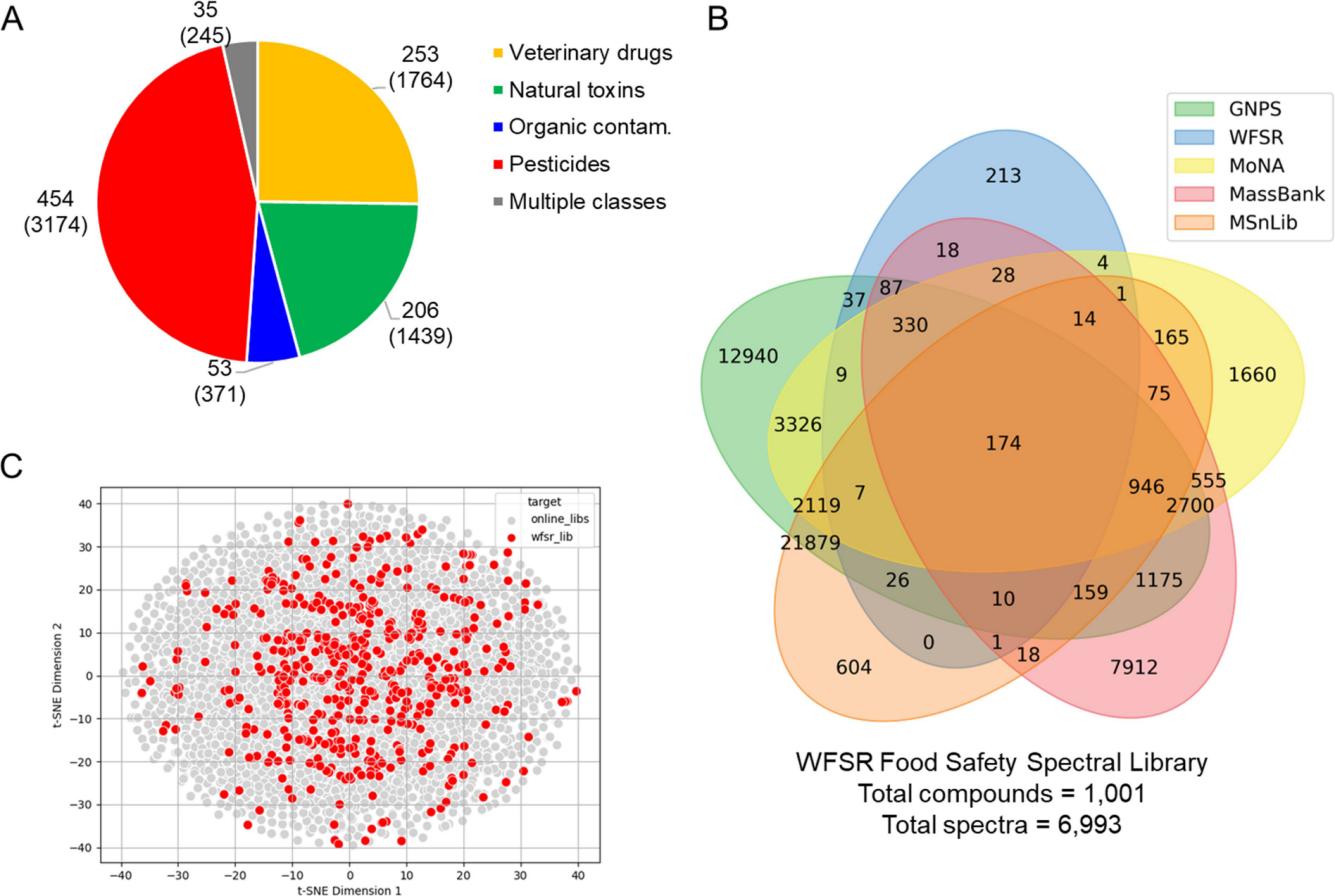
Library description and comparison with other open-access spectral libraries. (A) Number of compounds and spectra (values in parentheses) in the WFSR library, classified as veterinary drugs, natural toxins, organic contaminants, pesticides, or multiple classes. (B) Venn diagram showing the uniqueness and compounds overlap (based on planar InChIKey codes; stereochemistry omitted) between the WFSR, GNPS, MassBank, MoNA, and MSnLib libraries. (C) Visualization of the chemical space covered by the WFSR spectral library compared to aforementioned libraries.

The distribution of precursor ions revealed that most compounds in the library have molecular weights below 900 Da (Figure S2). A significant proportion of these fall within the *m*/*z* range of 265–340 and elute between 10 and 13 min (Figure S3). Notably, the compounds span the entire retention time range, showing a comprehensive coverage across all regions of the chromatographic space (Figure S3).

A comparison of planar InChIKey codes from the WFSR library with those in other open-access spectral libraries, GNPS, MassBank, MoNA, and MSnLib, revealed that 213 planar InChIKeys (representing 216 different compounds) are absent from these public repositories (Figure 1B). This highlights a significant gap in publicly available spectral data for food toxicants. The WFSR library contains 959 unique planar InChIKeys, representing only 1.68% of the total InChIKeys (56,979) in the combined online spectral libraries. Yet, 22.2% of the compounds in the WFSR library are not found in any of these databases. A full list of the 216 unique compounds is provided in Table S3 and their class distribution in Figure S4.

However, it is important to highlight that the absence of spectral data for a particular compound in public repositories should not be the only factor justifying the acquisition of new spectra. Although a compound might already have spectra available in the public domain, there is high heterogeneity in spectral quality and acquisition settings. Spectra acquired in low-resolution instruments are still common in online libraries, and there is no standardization in the collision energies used for fragmentation. For example, from the 746 matching compounds between the WFSR and the online libraries, only 580 (77.8%) have spectra available for more than five collision energies and 26 (3.5%) compounds have only one spectrum available. As the WFSR library includes spectral data acquired at seven different collision energies for the majority of the compounds, this increases the chances of annotation-relevant food toxicants in real samples even for compounds already available in online libraries, especially for those acquired on low-resolution instruments and under varied acquisition settings. Furthermore, the food toxicants in the WFSR library span diverse regions of the chemical space covered by the existing online libraries, as illustrated in Figure 1C.

Comparisons with the mzCloud library (www.mzcloud.org), using the full InChIKey codes, revealed that 362 compounds (36.2%) from our library are absent in this commercial resource, according to our search criteria. Of the 216 compounds unique to our library relative to the publicly available online repositories, 136 compounds are also absent in mzCloud. These results suggest that our spectral library addresses a significant gap in existing resources, as it provides novel and valuable data for a relevant number of compounds absent in public and commercial spectral libraries.

The spectral data of the WFSR Food Safety Mass Spectral Library were validated using multiple approaches. This included the analysis of QC samples, spectral clustering and library matching through GNPS,^5^ and machine learning-based spectral matching using MS2Query.^15^ These complementary methods established a robust framework to assess the reliability of the library spectra.

### Analysis of QC Mixtures

The analysis of the two QC samples, run at the beginning and at the end of each chromatographic sequence, demonstrated strong reproducibility in data acquisition. As illustrated in Figures S5–S10, the 34 compounds in the two standard mixtures consistently exhibited stable mass accuracy, retention times, and signal intensity across all 26 injection days. Notably, the retention time deviations were generally around 0.05 min (equivalent to 3 s, Figures S5 and S6), while the mass accuracy for all compounds remained within 2 ppm on all measurement days (Figures S7–S8). Furthermore, relative standard deviations of log_1__0_-transformed peak intensities ranged from 1.26 to 5.01% (Table S2), suggesting good reproducibility in instrument performance over time.

### Molecular Networking and Library Annotation in GNPS

Molecular networking in GNPS provided an overview of the similarities in HRMS/MS spectra between the library compounds (Figure 2). This approach enabled an assessment of spectral quality based on the clustering of molecular families, where compounds sharing similar spectra (indicative of structural similarities) are expected to group within the same subnetworks. This clustering pattern was observed for several chemical classes, as illustrated in Figure 2A for pyrrolizidine alkaloids, estrogenic steroids, ergot alkaloids, and sulfonamides, among others. This observation suggests that structural similarities in those compounds are represented by similarities in their HRMS/MS spectra. However, it is important to note that the four compound categories chosen in the present study (natural toxins, veterinary drugs, pesticides, and organic contaminants) do not represent accurate chemical classes. These broad categories were chosen to facilitate easier filtering for end-users working in a particular study field.

**Figure 2:**
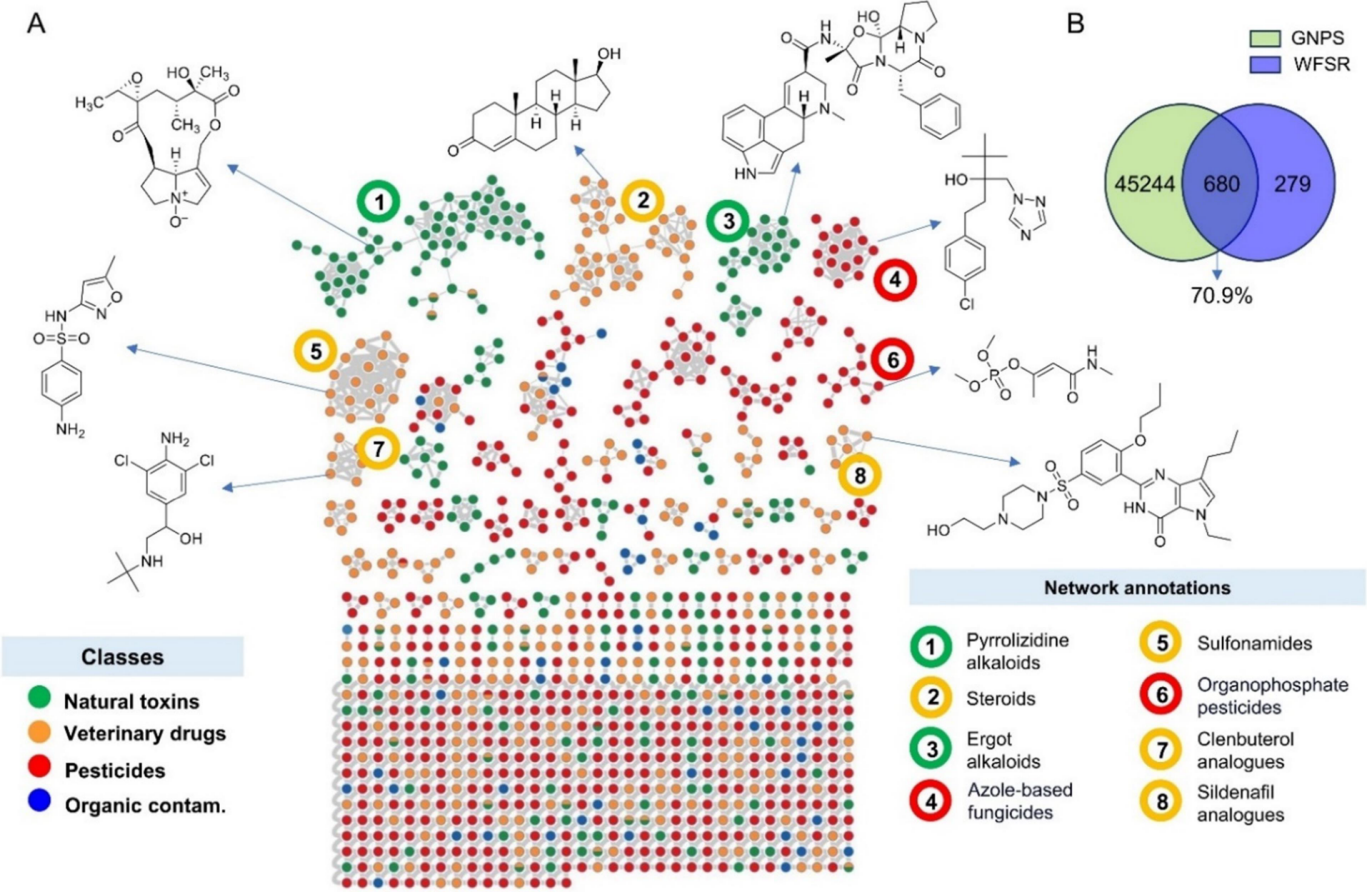
Molecular networking of the spectral data in the WFSR library. (A) Molecular network colored by the assigned compound classes showing representative structures from selected networks manually annotated. Nodes with different colors represent compounds belonging to multiple classes. (B) Venn diagram showing the uniqueness and compounds overlap (based on planar InChIKey codes; stereochemistry omitted) between the WFSR and the cleaned GNPS libraries.

From the 1001 compounds in our spectral library, GNPS annotated 560 by spectral matching using the modified cosine similarity metric.^5^ Of these annotations, 503 (50.2%) showed a matching planar InChIKey with our library (381 when full InChIKeys were considered instead). This indicates that approximately half of the compounds in our library were either absent from the GNPS reference libraries, incorrectly matched, or associated with low-quality spectra. To investigate this further, we first compared the planar InChIKeys in our library with those in the cleaned GNPS libraries (see methods for details). We found 680 matches (70.9%) between the two data sets (Figure 2B), suggesting that some compounds in the WFSR library may have been misannotated under the GNPS matching criteria (minimum of four matched peaks and a modified cosine score >0.7) or had a low-quality spectrum. However, this comparison should be interpreted cautiously, as the spectral libraries used in the GNPS library search differ in curation and metadata from the downloadable cleaned GNPS libraries used here.

Therefore, to further assess spectrum quality and annotation accuracy, we randomly selected 10 compounds not annotated by molecular networking that were present in both the WFSR and the cleaned GNPS libraries and had spectra acquired at NCE30. We selected compounds from the four assigned classes, keeping a similar proportion to our library data (Table 1). For each compound, we calculated the modified cosine score using the matchms package in Python 3.11 and generated mirror match plots of the spectrum pairs (Figures S11–S20). Of the 10 selected compounds, eight had a modified cosine score below the 0.7 threshold (Table 1).

**Table 1:** Ten Selected Compounds Shared between the WFSR and the Cleaned GNPS Libraries but Not Annotated by Molecular Networking, Including the Modified Cosine Score between Spectrum Pairs and Assigned Classes

InChIKey	modified cosine score	compound name	assigned class
ANVREEJNGJMLOV-UHFFFAOYSA-N	0.32	tris(4-isopropylphenyl) phosphate	organic contaminant
BULVZWIRKLYCBC-UHFFFAOYSA-N	0.98	phorate	pesticide
KUBCEEMXQZUPDQ-UHFFFAOYSA-N	0.27	hordenine^a^	natural toxin
NDNUANOUGZGEPO-UHFFFAOYSA-N	0.60	coniine	natural toxin
PJSFRIWCGOHTNF-UHFFFAOYSA-N	0.71	sulfadoxine	veterinary drug
LDLMOOXUCMHBMZ-UHFFFAOYSA-N	0.66	bixafen	pesticide
XRQHTUDGPWMPKX-UHFFFAOYSA-N	0.51	phorate sulfoxide	pesticide
WEBQKRLKWNIYKK-UHFFFAOYSA-N	0.11	demethon-*S*-methyl [M + Na]^+^ ^a^	pesticide
YREQHYQNNWYQCJ-UHFFFAOYSA-N	0.07	etofenprox [M + NH_4_]^+^ ^a^	pesticide
ZCGNOVWYSGBHAU-UHFFFAOYSA-N	0.07	favipiravir^a^	veterinary drug

a Compound absent in the MS2Query library.

Visual inspection of the spectrum pairs revealed significant differences in both the number and intensity of fragment ions. These discrepancies are primarily due to variations in instrumentation and acquisition settings, including the type of fragmentation method and overall spectrum quality. In the WFSR library, we standardized the use of HCD fragmentation, which operates at higher collision energies and generates more low *m*/*z* fragments compared to collision-induced dissociation (CID). In contrast, GNPS spectra originate from multiple instruments employing various fragmentation methods, such as CID in ion traps, with differing collision energies and fragmentation conditions. For instance, the GNPS spectrum of tris­(4-isopropylphenyl) phosphate (Figure S11), a neurotoxic environmental contaminant, showed fragments only above 320 *m*/*z*, while our library contained more informative fragments and a better quality spectra, illustrating the relevance of acquiring high-quality spectral data even for compounds already available in public repositories. Similarly, the GNPS spectra of coniine, a piperidine alkaloid, illustrated in Figure S14, displayed the precursor ion only, while the spectra present in our library contained more informative diagnostic fragments. The spectra of sulfadoxine (Figure S15) and etofenprox (Figure S17) exhibited a large number of low-intensity noisy peaks in GNPS, further emphasizing the need for consistent and optimized acquisition protocols. In contrast, phorate, an organophosphate pesticide, which had the highest modified cosine score of 0.98 (Figure S12), yielded no results when searched individually in GNPS2 (https://library.gnps2.org/). This confirms that the spectral libraries used in the molecular networking-based library search differ from the cleaned GNPS libraries available for download, as expected.

### Spectral Matching in MS2Query

To further assess the spectral quality of our library, we performed spectral matching in MS2Query^15^ v1.5.3, using its matchms-cleaned library that contains curated spectra from GNPS, MassBank, MoNA, and MSnLib.^12^ For this, the WFSR library was first split by collision energies and the spectrum collection for each energy were treated as “sample sets”. Annotation was performed by spectral similarity using the Spec2Vec^16^ and MS2Deepscore algorithms.^17^ To determine the percentage accuracy, the software annotation obtained for each spectrum in the sample set, represented by its planar InChIKey identifier, was compared to the library InChIKey.

As observed in Figure 3A, MS2Query yielded a higher annotation accuracy compared to GNPS-based molecular networking. Spectra acquired at NCE30 had the highest number of correct annotations with 634 compounds (63.4%), while spectra acquired at higher collision energies showed decreasing percentages (Figure 3A). Notably, annotation accuracy varied not only with collision energy but also across different compound classes. As shown in Figure 3B, organic contaminants had the lowest percentage of correct annotations, at 56.6%. This suggests that these compounds are likely underrepresented in the positive mode MS2Query libraries, which is expected since many of them preferentially ionize in negative mode.

**Figure 3:**
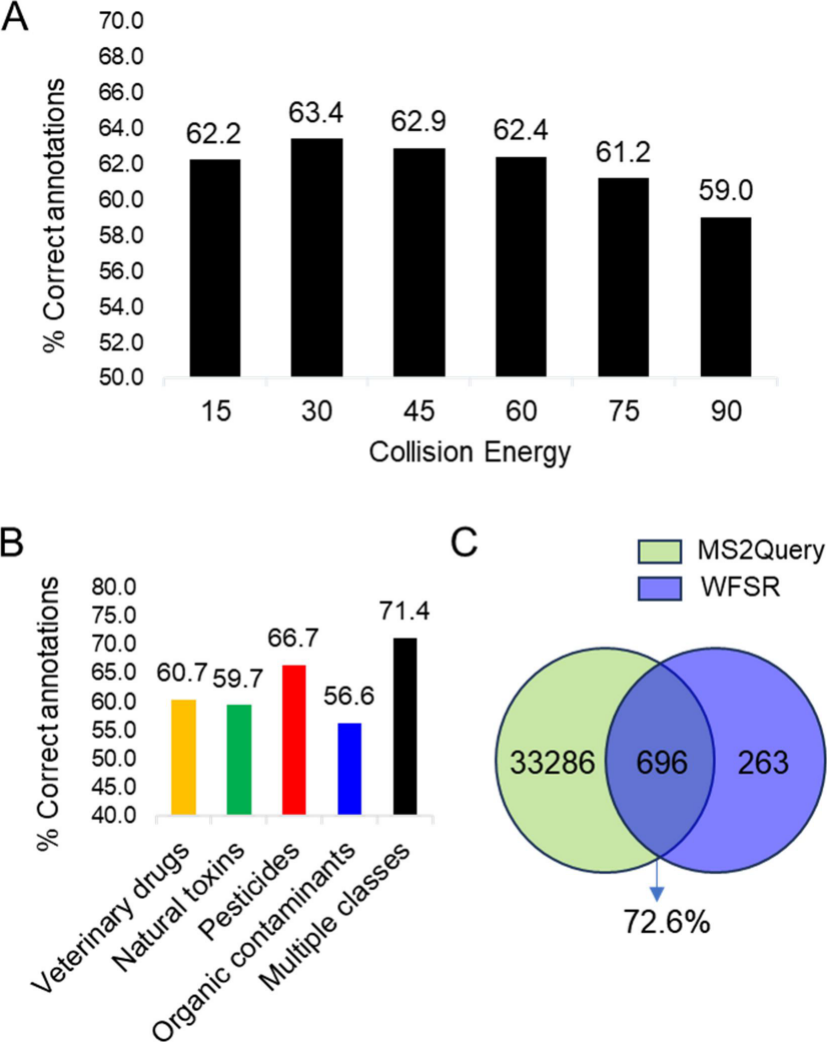
Annotation accuracy in the software MS2Query by collision energy (A) and assigned classes (B). Venn diagram (C) showing the uniqueness and compounds overlap (based on planar InChIKey codes; stereochemistry omitted) between the WFSR and the MS2Query libraries.

Further comparisons revealed that 696 (72.6%) planar InChIKeys in our library are shared with the MS2Query libraries (Figure 3C), while 263 (27.4%) are unique. Considering only the shared compounds between both data sets, MS2Query correctly identified 86.5% for the NCE30 sample set. This result highlights not only the high accuracy of MS2Query annotations but also validates the reliability of our spectral data. For instance, an individual search for the 10 non-annotated compounds by molecular networking (Table 1) showed correct annotations for six of them by MS2Query. Further interrogation of the four incorrect annotations indicated that the WFSR library spectra of demethon-*S*-methyl and etofenprox was obtained from the sodium and ammonium adducts, respectively, while the spectra available online for these compounds were obtained from protonated molecules.

## Conclusions

Dedicated and open-access spectral libraries focused on food toxicants are a critical yet largely unmet need in food safety, with the potential to serve as a foundation for the rapid and accurate identification of harmful substances. The WFSR Food Safety Mass Spectral Library addresses this gap by offering a comprehensive, freely accessible collection of tandem mass spectral data on food contaminants, residues, and related hazardous compounds. This library comprises 6993 manually curated HRMS/MS in positive ionization mode from 1001 compounds.

Although the number of compounds in the WFSR library represents only a small fraction compared to those available in public libraries (GNPS, MassBank, MoNA, and MSnLib), we found that 22.2% of these compounds are absent from those larger repositories. This highlights the need to improve the representation of food toxicants in publicly available spectral databases. Furthermore, we showed that, for certain compounds, the WFSR library includes spectra acquired at additional fragmentation energies not available online and in some cases provides spectra of higher quality. The WFSR library also offers complementary data for selected compounds by including spectra generated using an additional LC-HRMS instrument (Orbitrap IQ-X) to the online available data.

Technical validation through spectral matching using molecular networking enabled the mapping of the chemical space covered by the acquired spectra, revealing clustering patterns consistent with certain chemical classes. Spectral annotation using MS2Query software showed annotation rates of 86% for compounds shared between the MS2Query and WFSR libraries, further validating the reliability of our spectral data.

We expect that by openly sharing the WFSR library in different formats and repositories, researchers will be able to integrate it into their data analysis workflows using licensed software (e.g., Compound Discoverer) and open-access tools (such as GNPS and MS2Query). This will enable high-throughput screening and enhance food safety monitoring across academic, industrial, and regulatory settings. Additionally, the library provides a valuable resource for developing more robust machine learning algorithms to improve annotation rates for food safety hazards.

The WFSR Food Safety Mass Spectral Library is an ongoing project at Wageningen Food Safety Research, with regular updates and expansions. Additional spectral data are currently being acquired to expand the positive mode coverage and to include compounds that ionize in negative mode. This expansion is particularly valuable, as spectra acquired in negative ionization mode remain underrepresented in public databases. It also opens new possibilities for cross-ionization mode comparisons and prediction of chemical similarity based on tandem mass spectra.^18^ Researchers are encouraged to contact the corresponding author for access to the latest version and to explore opportunities for collaboration.

## Supplementary Material









## Data Availability

The final library containing 1001 compounds and 6993 HRMS/MS spectra is available as .db and .msp formats through a dedicated website hosted at Wageningen Food Safety Research (accessible at https://www.wur.nl/en/research-results/research-institutes/food-safety-research/show-wfsr/food-safety-mass-spectral-library.htm). These data formats allow our spectral library to be directly implemented in data-processing workflows of different software, including commercial software like Compound Discoverer (Thermo Fisher Scientific). Additionally, the entire spectral library in .mgf format is also publicly available through the GNPS repository to facilitate community reuse. Each HRMS/MS spectrum was assigned an individual accession number ranging from CCMSLIB00013933782 to CCMSLIB00013940774. The spectral collection is available for download under the name WFSR Food Safety Mass Spectral Library at https://external.gnps2.org/gnpslibrary. The molecular network (cytoscape file and associated data set) shown in Figure 2 can accessed at ftp://massive-ftp.ucsd.edu/v10/MSV000098571/, and the GNPS results can be accessed at https://gnps.ucsd.edu/ProteoSAFe/status.jsp?task=00a0181c5d464208b330211cdf46b336. The library upload in the GNPS repository will facilitate the automatic annotation of food toxicants in various matrices through molecular networking and related workflows, helping identify structurally related analogues in future studies.

## Notes

(Table S1) Complete list of the reference standard names, their molecular formulas, precursor ion (*m*/*z*) values, retention times, SMILES, and InChIKeys (XLSX)
(Table S2) List of compounds in each QC mixture, including molecular formulas, precursor ion (*m*/*z*) values, and retention times (XLSX)
(Table S3) List of compounds unique to the WFSR library in relation to the online libraries (GNPS, MassBank, MoNA, and MSnLib) (XLSX)
(Figure S1) General workflow followed for library building, curation, and technical validation; (Figures S2 and S3) distribution of precursor ions and retention time values; (Figure S4) class distribution of the 216 compounds unique to the WFSR library; (Figures S5–S10) deviation in retention time, ppm error, and log_10_ intensity for each compound in the QC mixtures injected on across all 26 injection days; (Figures S11–S20) mirror match plots for the 10 compounds selected for validation with shared spectra between the WFSR and cleaned GNPS libraries (PDF)
