# Supplementary material for: Creation of an Open-Access High-Resolution Tandem Mass Spectral Library of 1000 Food Toxicants
Source: Anal Chem. 2025 Oct 10;97(43):23822–30. doi: 10.1021/acs.analchem.5c03020 (PMC12590464; doi:10.1021/acs.analchem.5c03020)
Supplement: Supplementary file 4 [file ac5c03020_si_004.pdf]

Supporting Information

Creation of an Open Access High-Resolution Tandem Mass Spectral Library of 1,000 Food Toxicants

Federico Padilla-González*, Serena Rizzo, Caroline Dirks, Wout Bergkamp, Sjors Rasker, Ivan Aloisi

Wageningen Food Safety Research, Wageningen University & Research, Akkermaalsbos 2, 6708 WB, Wageningen, The Netherlands

* Email: federico.padillagonzalez@wur.nl

Table of Contents

Figure S1. General workflow containing the main steps followed for library building, curation and technical validation of the WFSR spectral library. 2

Figure S2. Distribution of precursor ions in the library, with the dark grey highlight, to indicate the region with the highest density of spectra. 3

Figure S3. Distribution of retention time (Rt) values across the library, with the range of 10.4–12.8 minutes highlighted, to indicate the interval with the highest density of spectra. 3

Figure S4. Class distribution of the 216 compounds unique to the WFSR library in relation to the online libraries (GNPS, MassBank, MoNA and MSnLib). 3

Figure S5. Retention time deviation of the 18 compounds (represented by individual lines) in the standard mixture QC1 injected in each measurement day. 4

Figure S6. Retention time deviation of the 16 compounds (represented by individual lines) in the standard mixture QC2 injected in each measurement day. 4

Figure S7. Ppm error of the 18 compounds (represented by individual lines) in the standard mixture QC1 injected in each measurement day. 5

Figure S8. Ppm error of the 16 compounds (represented by individual lines) in the standard mixture QC2 injected in each measurement day. 5

Figure S9. Intensity deviation (Log_{10}) of the 18 compounds (represented by individual lines) in the standard mixture QC1 injected in each measurement day. 6

Figure S10. Intensity deviation (Log_{10}) of the 16 compounds (represented by individual lines) in the standard mixture QC2 injected in each measurement day. 6

Figure S11. Mirror match plot for the compound Tris(4-isopropylphenyl) phosphate (IPPP) between the MS2 spectra in the WFSR (top) and GNPS (bottom) libraries. 7

Figure S12. Mirror match plot for the compound Phorate between the MS2 spectra in the WFSR (top) and GNPS (bottom) libraries. 7

Figure S13. Mirror match plot for the compound Hordenine between the MS2 spectra in the WFSR (top) and GNPS (bottom) libraries. 8

Figure S14. Mirror match plot for the compound Coniine between the MS2 spectra in the WFSR (top) and GNPS (bottom) libraries. 8

Figure S15. Mirror match plot for the compound Sulfadoxine between the MS2 spectra in the WFSR (top) and GNPS (bottom) libraries. 9

Figure S16. Mirror match plot for the compound Bixafen between the MS2 spectra in the WFSR (top) and GNPS (bottom) libraries. 9

Figure S17. Mirror match plot for the compound Phorate sulfoxide between the MS2 spectra in the WFSR (top) and GNPS (bottom) libraries. 10

Figure S18. Mirror match plot for the compound Demethon-S-methyl [M+Na]+ between the MS2 spectra in the WFSR (top) and GNPS (bottom) libraries. 10

Figure S19. Mirror match plot for the compound Etofenprox [M+NH4]+ between the MS2 spectra in the WFSR (top) and GNPS (bottom) libraries. 11

Figure S20. Mirror match plot for the compound Favipiravir between the MS2 spectra in the WFSR (top) and GNPS (bottom) libraries. 11

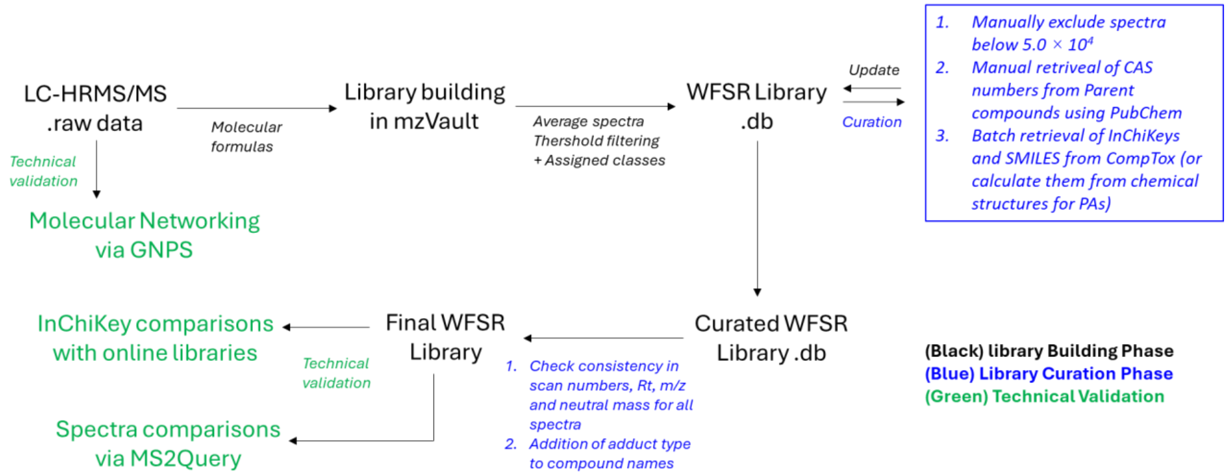

Figure S1. General workflow containing the main steps followed for library building, curation and technical validation of the WFSR spectral library.

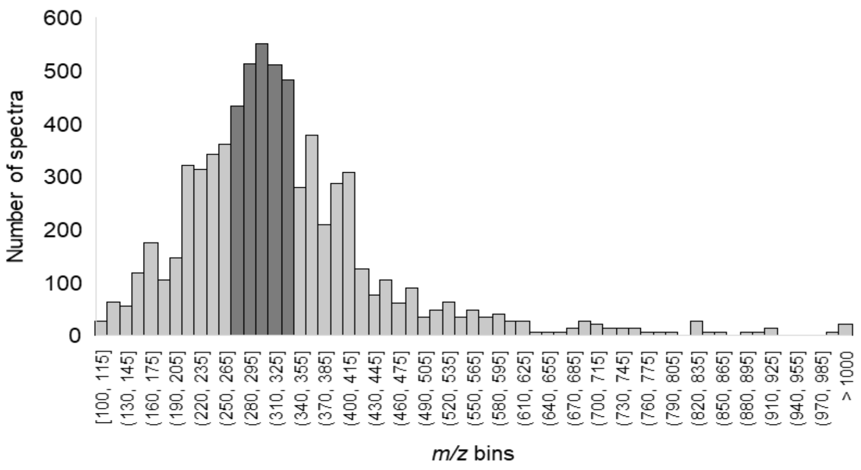

Figure S2. Distribution of precursor ions in the library, with the dark grey highlight, to indicate the region with the highest density of spectra.

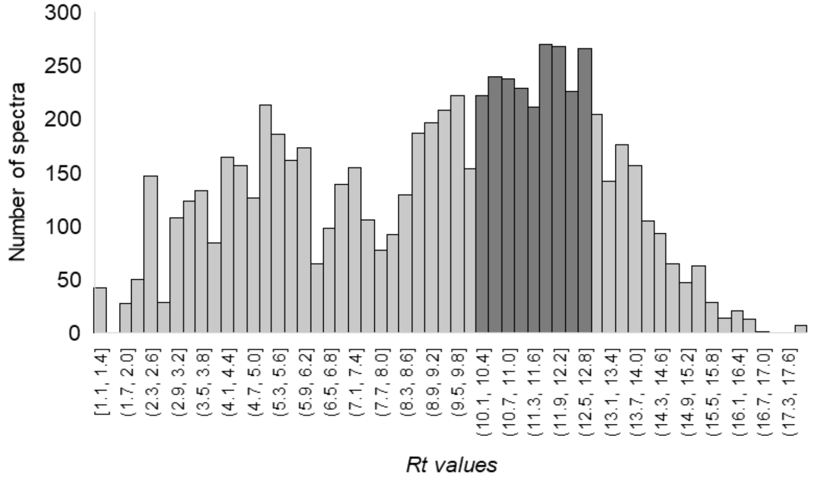

Figure S3. Distribution of retention time (Rt) values across the library, with the range of 10.4–12.8 minutes highlighted, to indicate the interval with the highest density of spectra.

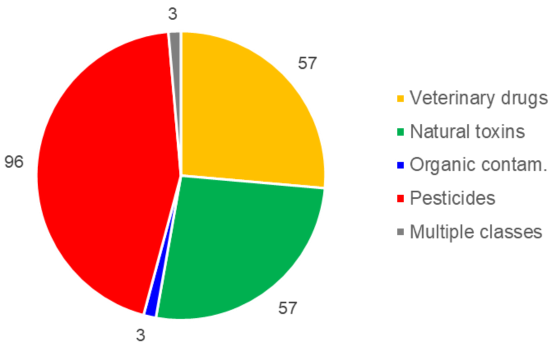

Figure S4. Class distribution of the 216 compounds unique to the WFSR library in relation to the online libraries (GNPS, MassBank, MoNA and MSnLib).

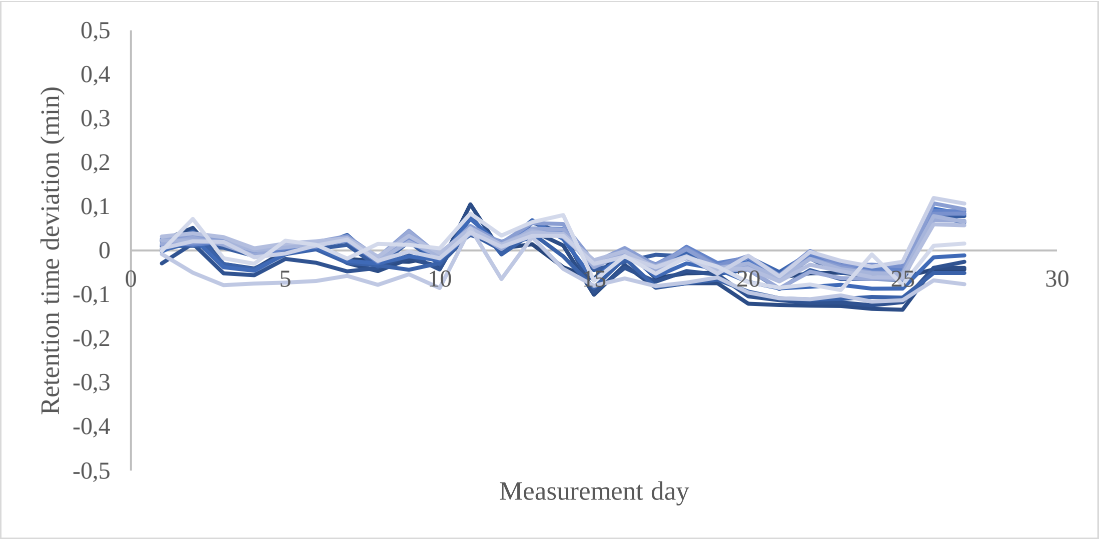

Figure S5. Retention time deviation of the 18 compounds (represented by individual lines) in the standard mixture QC1 injected in each measurement day.

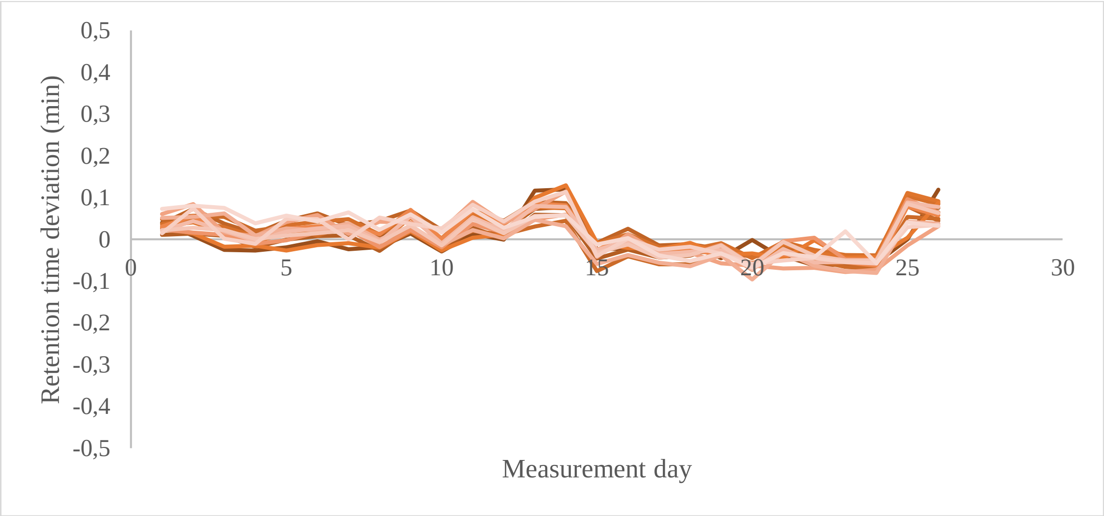

Figure S6. Retention time deviation of the 16 compounds (represented by individual lines) in the standard mixture QC2 injected in each measurement day.

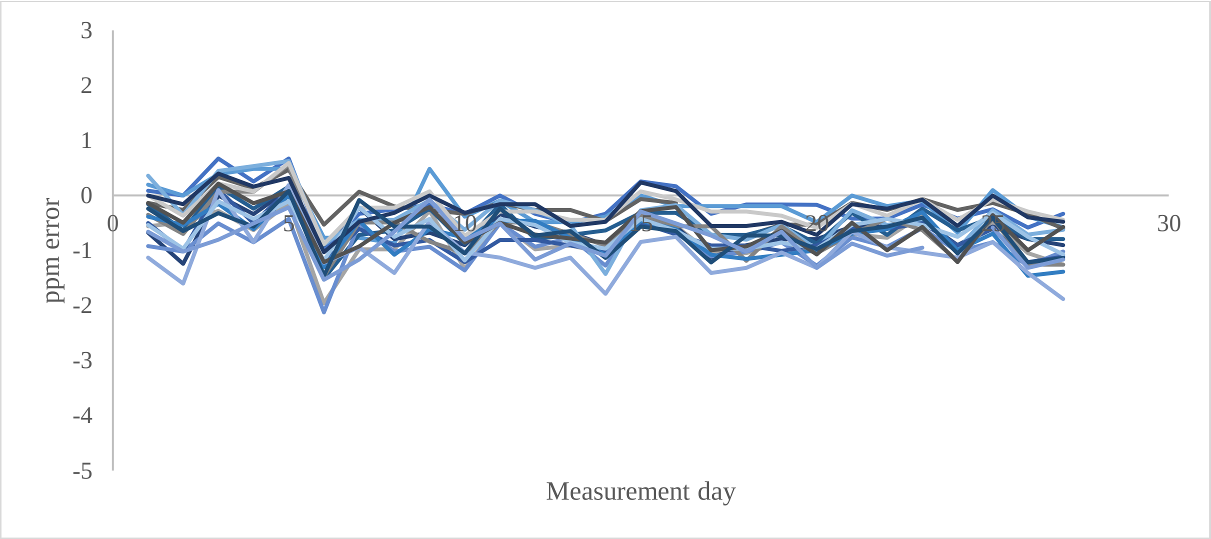

Figure S7. Ppm error of the 18 compounds (represented by individual lines) in the standard mixture QC1 injected in each measurement day.

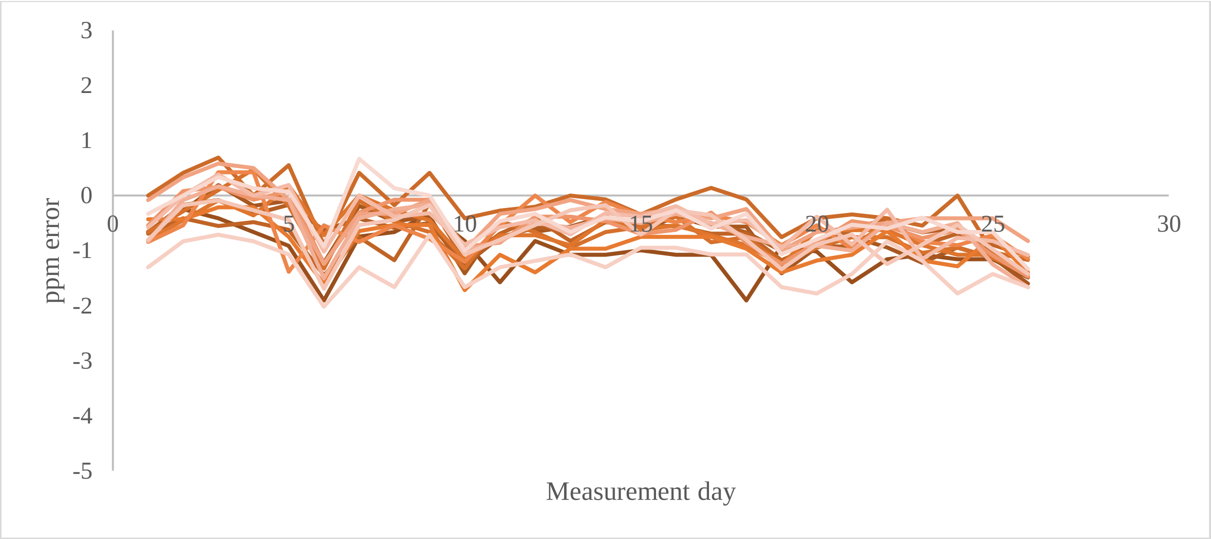

Figure S8. Ppm error of the 16 compounds (represented by individual lines) in the standard mixture QC2 injected in each measurement day.

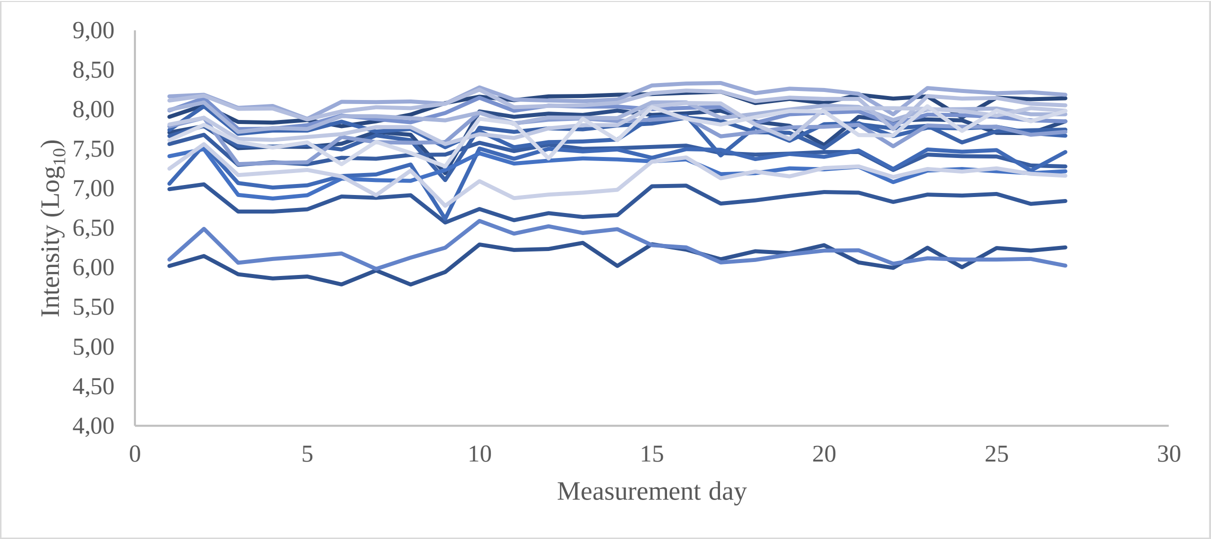

Figure S9. Intensity deviation (Log_{10}) of the 18 compounds (represented by individual lines) in the standard mixture QC1 injected in each measurement day.

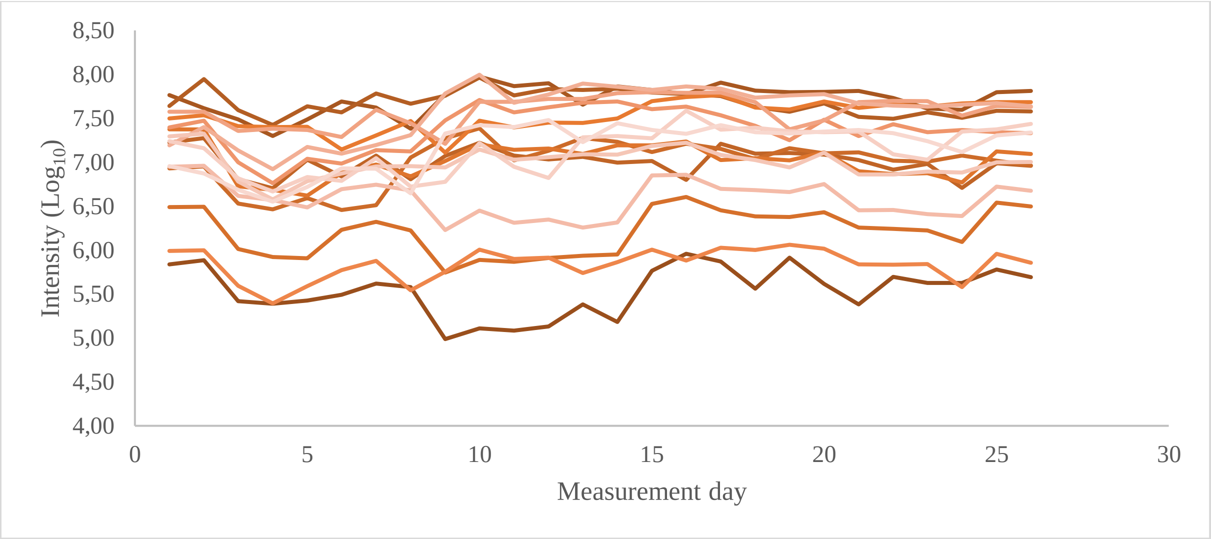

Figure S10. Intensity deviation (Log_{10}) of the 16 compounds (represented by individual lines) in the standard mixture QC2 injected in each measurement day.

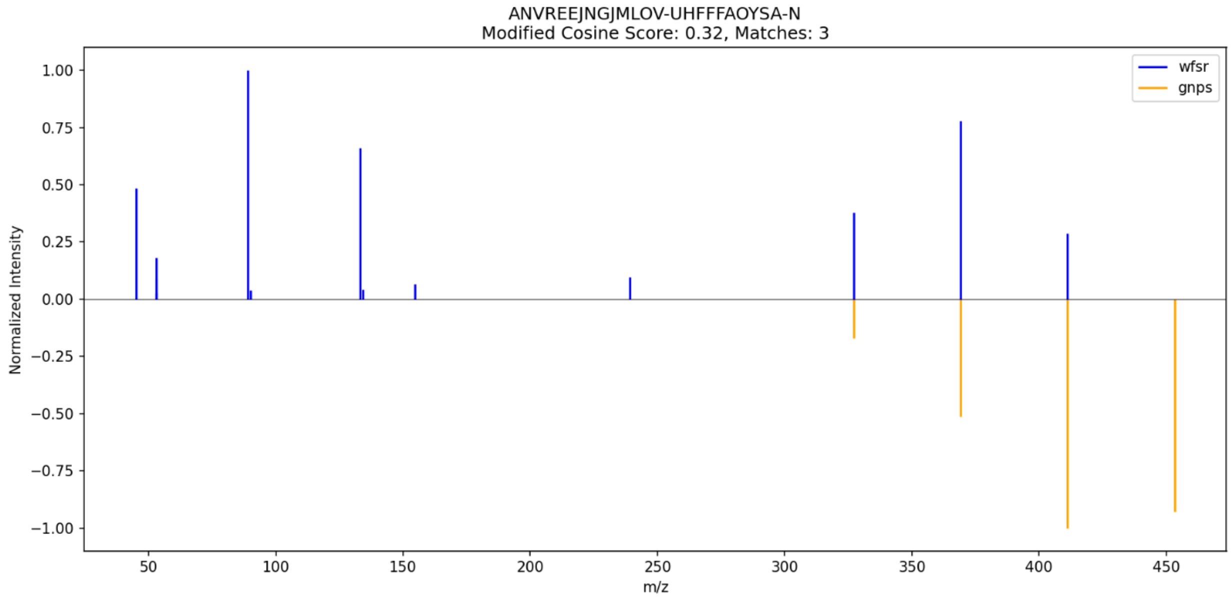

Figure S11. Mirror match plot for the compound Tris(4-isopropylphenyl) phosphate (IPPP) between the MS2 spectra in the WFSR (top) and GNPS (bottom) libraries.

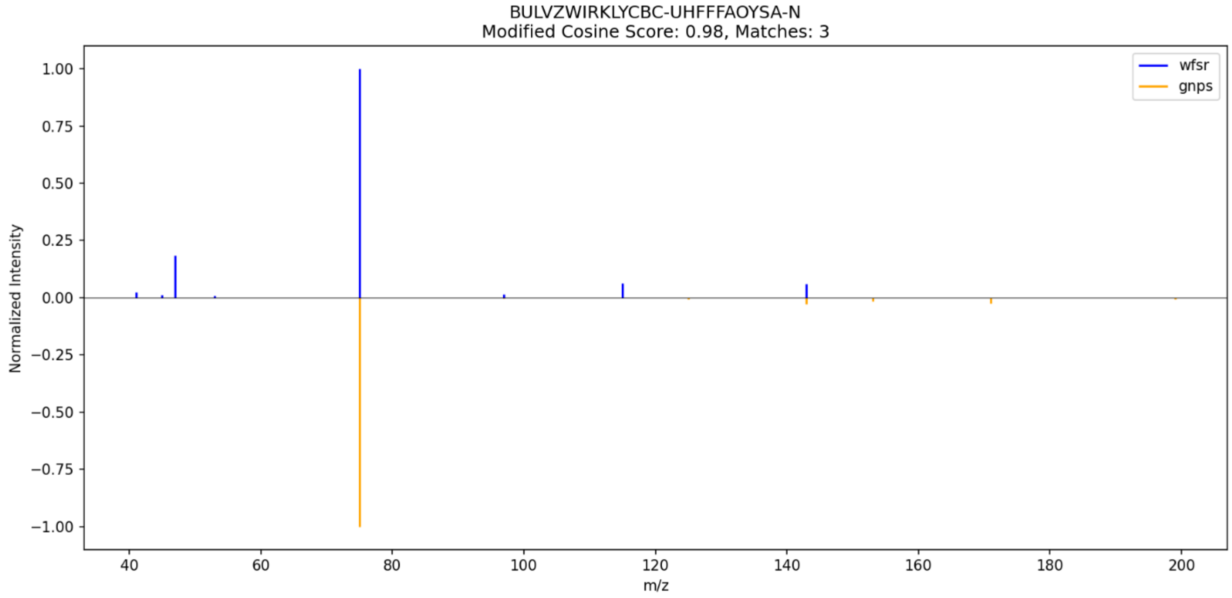

Figure S12. Mirror match plot for the compound Phorate between the MS2 spectra in the WFSR (top) and GNPS (bottom) libraries.

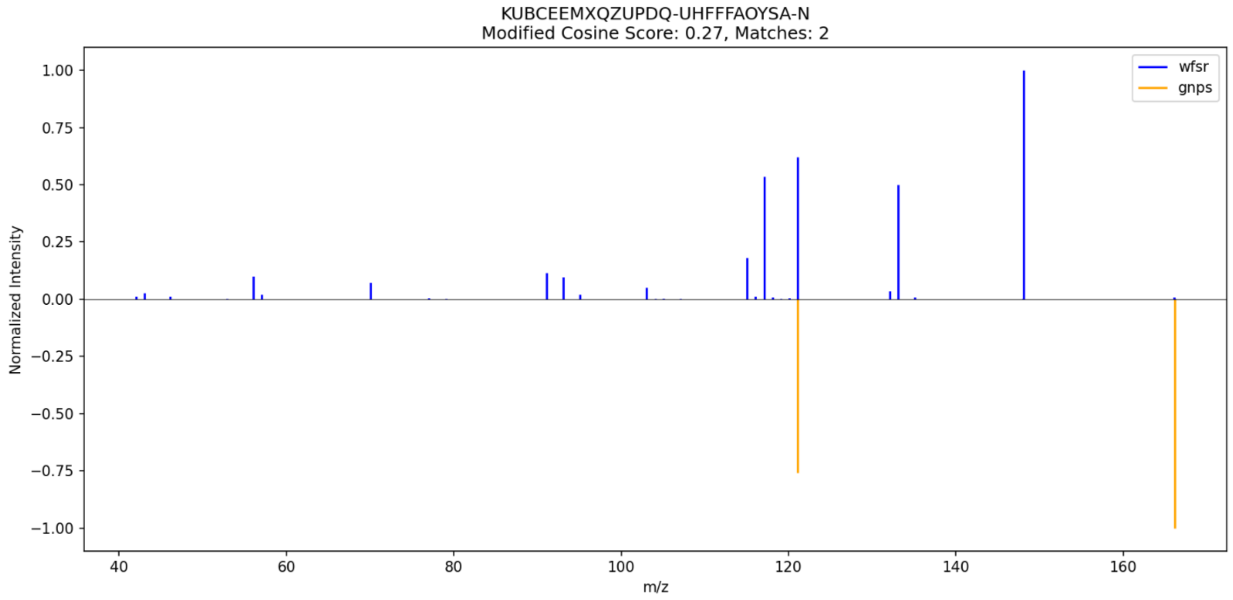

Figure S13. Mirror match plot for the compound Hordenine between the MS2 spectra in the WFSR (top) and GNPS (bottom) libraries.

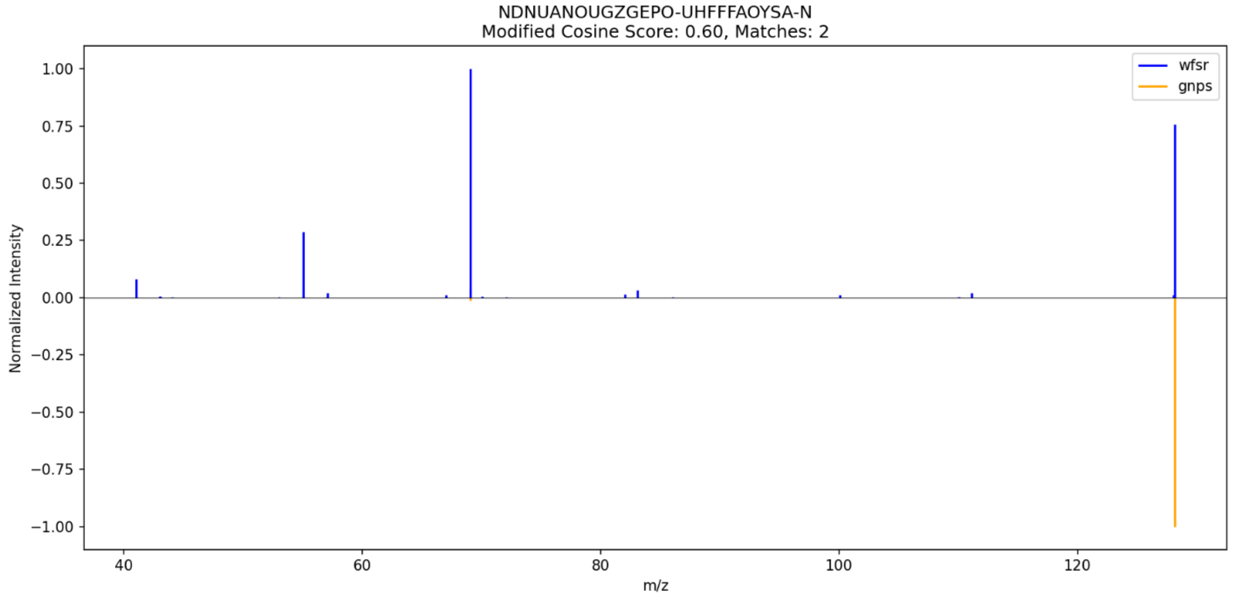

Figure S14. Mirror match plot for the compound Coniine between the MS2 spectra in the WFSR (top) and GNPS (bottom) libraries.

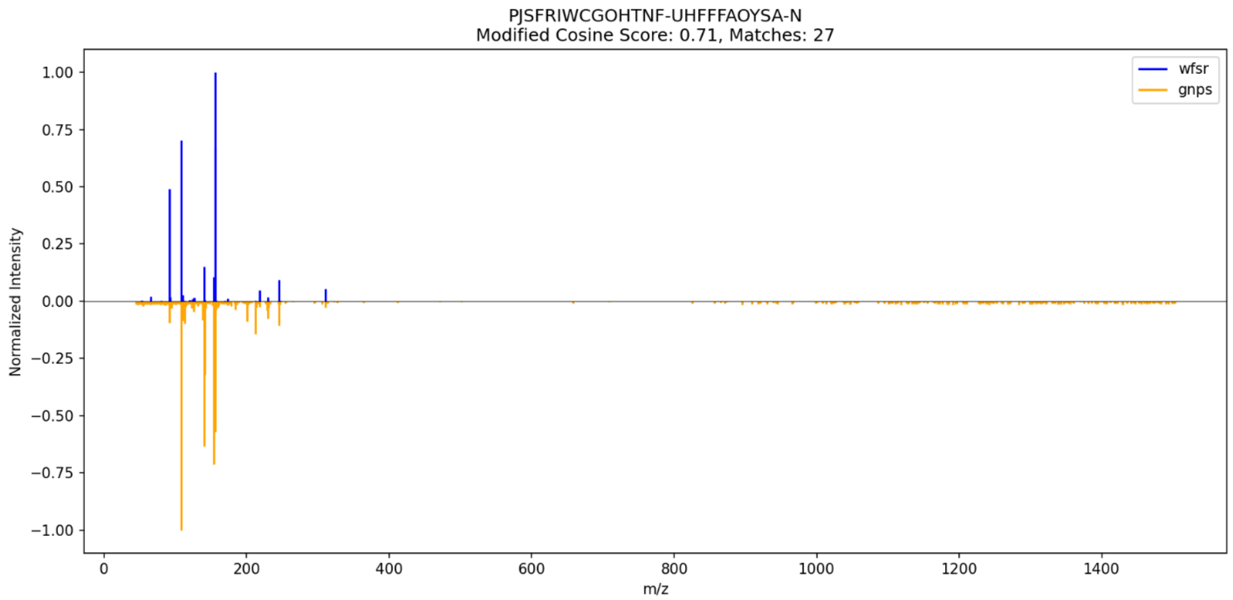

Figure S15. Mirror match plot for the compound Sulfadoxine between the MS2 spectra in the WFSR (top) and GNPS (bottom) libraries.

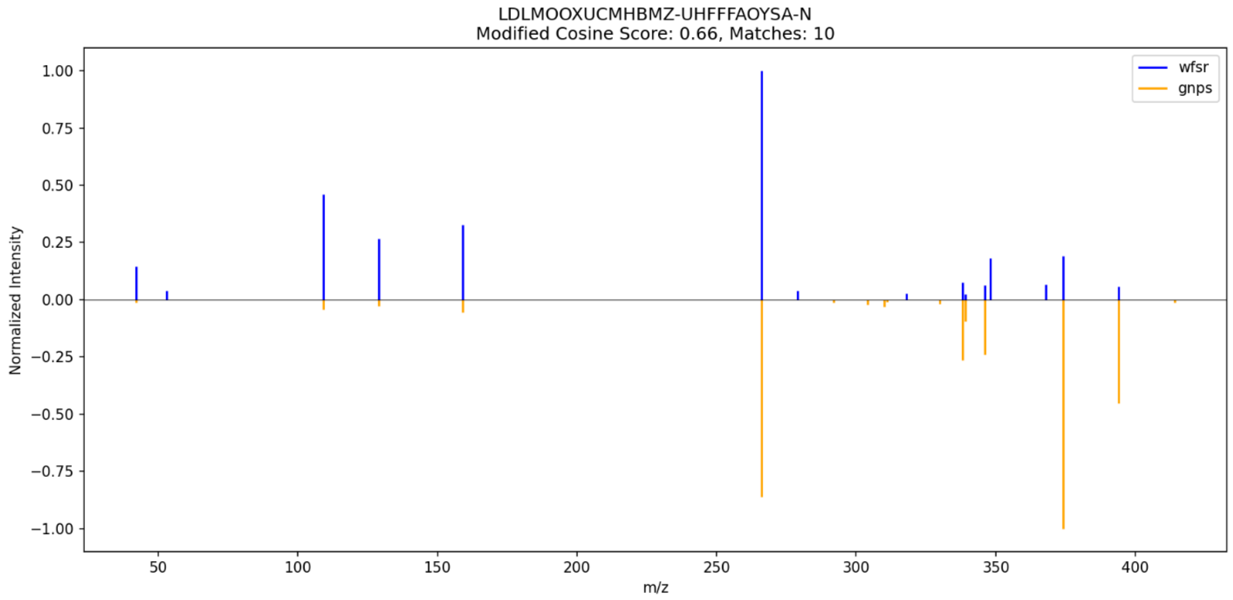

Figure S16. Mirror match plot for the compound Bixafen between the MS2 spectra in the WFSR (top) and GNPS (bottom) libraries.

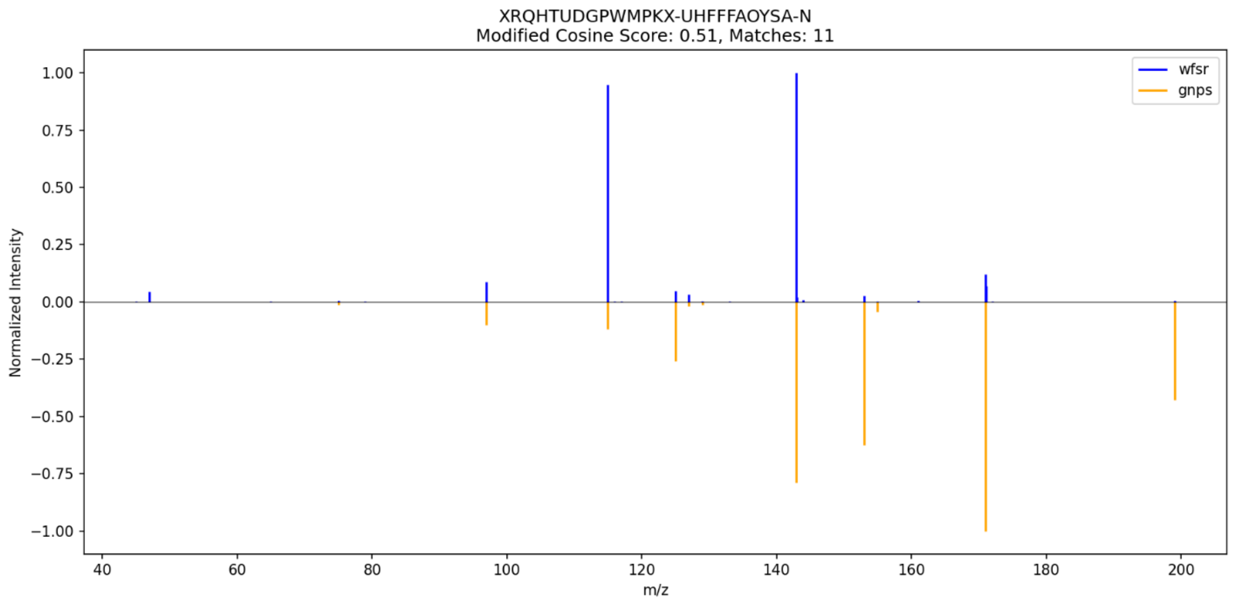

Figure S17. Mirror match plot for the compound Phorate sulfoxide between the MS2 spectra in the WFSR (top) and GNPS (bottom) libraries.

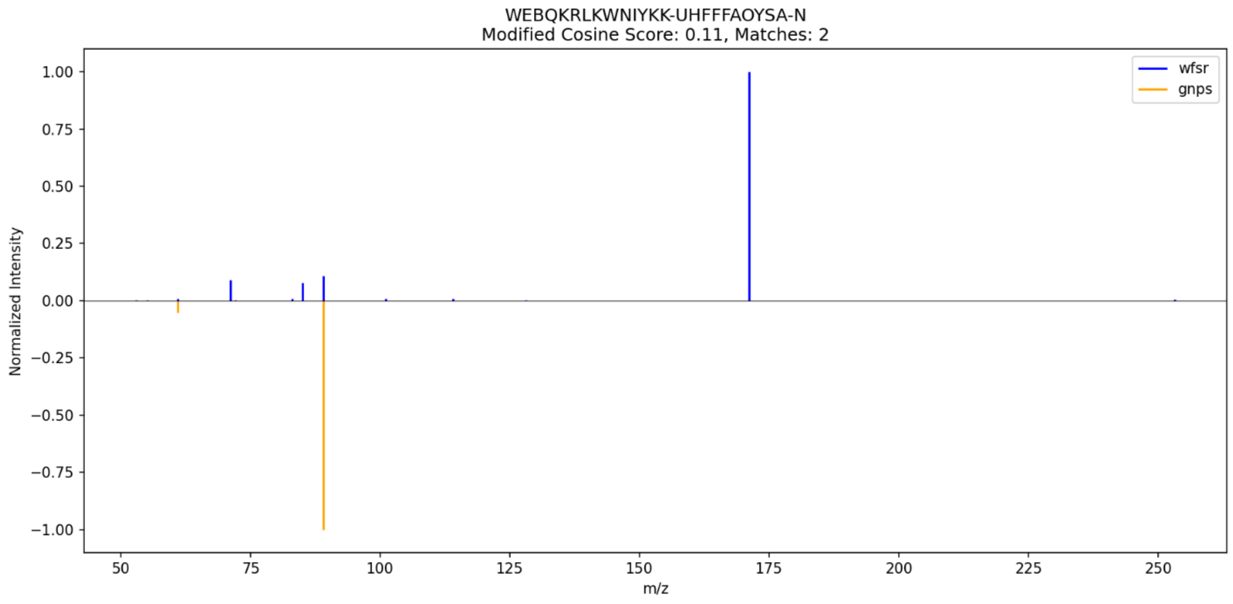

Figure S18. Mirror match plot for the compound Demethon-S-methyl [M+Na]+ between the MS2 spectra in the WFSR (top) and GNPS (bottom) libraries.

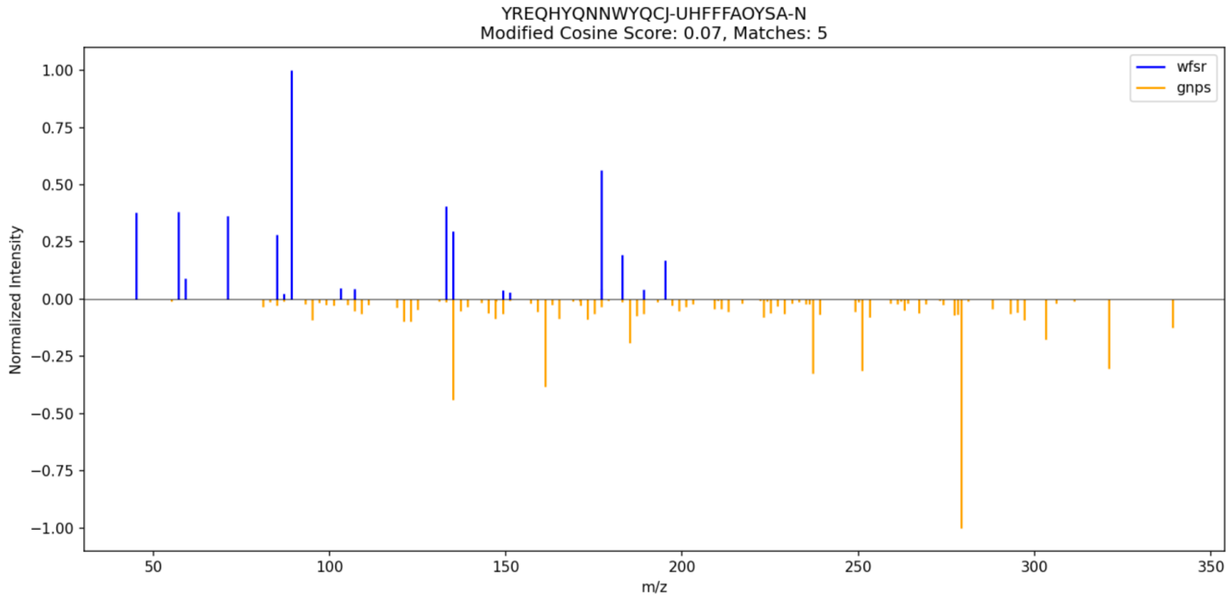

Figure S19. Mirror match plot for the compound Etofenprox [M+NH4]+ between the MS2 spectra in the WFSR (top) and GNPS (bottom) libraries.

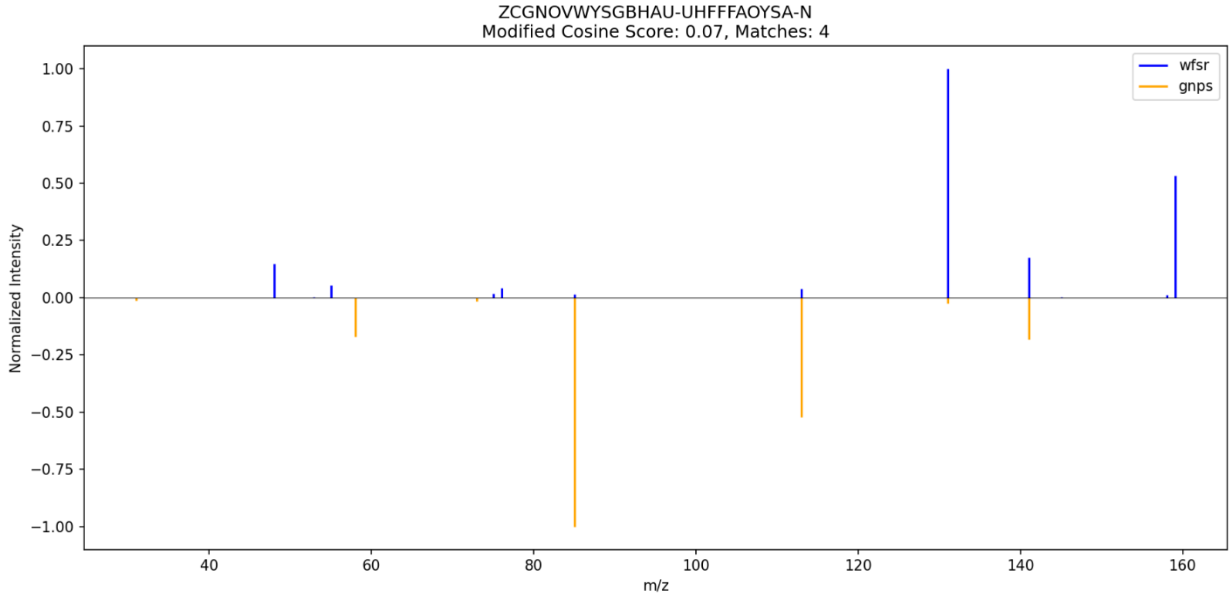

Figure S20. Mirror match plot for the compound Favipiravir between the MS2 spectra in the WFSR (top) and GNPS (bottom) libraries.
